# Integrative Behavioral Health (IBH) Model at the Intersections of the Philippine Mental Health Law, Education, and Policy: For COVID-19 Recovery and Beyond

**DOI:** 10.18178/ijlt.7.2.142-153

**Published:** 2021-06

**Authors:** Ruel R. Billones, Sam Aquino, Rey Jan Pusta, Marie Joyce Victolero-Tupas

**Affiliations:** NINR, National Institutes of Health, Bethesda, MD USA; Department of Psychology, School of Arts and Sciences, Ateneo de Davao University, Davao City, Philippines; Department of Psychology, School of Arts and Sciences, Ateneo de Davao University, Davao City, Philippines; Department of Psychology, School of Arts and Sciences, Ateneo de Davao University, Davao City, Philippines

**Keywords:** emancipatory education, Philippine Mental Health Law, symptom science, Filipino psychology, integrative behavioral health psychology, COVID-19

## Abstract

The COVID-19 pandemic has impacted the mental health of Philippine citizens. The authors propose the Integrative Behavioral Health (IBH) model to help facilitate the country’s eventual recovery from a health psychology perspective. Findings were integrated from a faculty consultation from a private university’s psychology department, a literature review, and a survey of students who are learning online. The survey results revealed that living with family members negatively correlated with readiness to learn online, *r* = −.37, *p* < .05. Further research is needed. Furthermore, combining themes gathered from the consultation, literature review, and variables used from the survey served as anchor words for the IBH model: 1. Emancipatory Education; 2) Filipino Psychology; 3) Contextualization; 4.) Philippine Mental Health Law; 5). Symptom Science; and 6) Social Determinants of Health (SDH). The constructs were implemented into an online health psychology course. The proposed curriculum design provides for an effective mental health response towards post-pandemic recovery.

## I. INTRODUCTION

The COVID-19 pandemic necessitates a multi-disciplinary approach to address the total health, including the Filipino citizenry’s mental health. It has been predicted that mental health issues will continue to be on the rise as a result of the pandemic [1], [2]. It requires multi-disciplinary, national, and international collaborations [3] and programs to recover from these mental health concerns [4]. Health psychology as a discipline that applies an evidence-informed model needs to be created to effectively respond to the country’s mental health issues today [5]. A proposed Integrative Behavioral Health (IBH) Model serves as a roadmap to navigate the health psychology’s role in the mental health recovery and healing of the Filipinos.

When the pandemic hit the country in the beginning of February 2020, no treatment was in sight. Behavior was the only option to mitigate the spread of the virus. [6] Enhanced Community Quarantine (ECQ) was enforced. Social distancing, shutting down of social places, were some of the behavioral interventions to mitigate the spread of the virus. In March 2020, major cities of the country like Manila, Cebu and Davao sustained a rapid increase in number of COVID-19 cases. In August of 2020, five months later, positive cases in these cities continue to surge; hence, stricter guidelines such as ECQ were enforced. ECQ, as a form of behavioral intervention, endangered the slum dwellers of the city and the citizens from the lower economic income bracket. One of the government’s challenges was related to the opening of schools, whether face to face instructions, hybrid approach, or completely online classrooms mode of delivery was to be implemented. The online learning approach raised mental health concerns among young students involved in purely online learning [7] There is no consolidated and national comprehensive program of the government to meet this growing mental health concern of the country [8]. Other countries like China [9] and England [10] have the clinical health psychologists play significant roles in designing psychological emergency manuals as national guidelines in the delivery of mental health care services for its citizens. The authors identified that one of the sources of the gaps is the lack of an implementation model that bridges the gaps between health psychology as a discipline and its ability to address the mental health needs of the Filipinos.

### A. The Philippine Mental Health Law

Countries all over the world have started promulgating the role of mental health in the holistic care of people [11]. To facilitate the development of national programs for mental wellbeing, the World Health Organization released an Assessment of Mental Health Systems (WHO-AIMS) focusing on policy and legislative framework, mental health services, mental health in primary care, human resources, education of the public at large, and monitoring and research [12]. Data showed that the Philippines’ ratio of mental health workers is as low as 2–3 per 100,000 population. The Philippines’ available mental health resources include 46 outpatient facilities, four community residential facilities, two tertiary care psychiatric hospitals with 4,200 and 500-bed capacity, and 12 smaller satellite hospitals [13].

The WHO-AIMS found the Filipino’s value for literacy, education, and professional development to develop mental health programs in the country. Academic institutions competently train mental health professionals, including psychiatrists, psychologists, nurses, social workers, and allied mental health professionals. However, there is still a wide discrepancy between the mental health professions and patient ratio and the implementation of mental health care delivery to the grassroots of Philippine society. With the current Philippine Mental Health Law in place, standardizing the training of professionals like health psychologists that can cascade to delivery of their mental health care to the grassroots is urgent and necessary during this time of the pandemic.

### B. Sikolohiyang Filipino (Filipino Psychology)

There is a vacuum of indigenous thinking in health psychology. The vacuum is filled with colonized paradigms that lack appreciation of Filipinos’ cultural context of mental health. Through the works of Dr. Virgilio G. Enriquez of the University of the Philippines, Filipino Psychology (FP) was established as an indigenous science [14]. Pioneers of FP assert that the colonialist system has “*miseducated*” the Filipino. Thus, Sikolohiyang Filipino is a movement towards a “*malaya*” (liberated) and “*mapagpalayang*” (liberating) system of thought.

Sikolohiyang Filipino is characterized as a science that is both liberated (malaya) and liberating (mapagpalaya). It is liberated because it advocates for independent thinking grown from grassroots experience. It is liberating because it advocates to represent the voice and interest of the oppressed sectors of society. The etic approach of conceptualizing local phenomena using a foreign lens often misses important cultural elements of the experience. The Sikolohiyang Filipino movement advocates for the use of an emic approach in examining local phenomena. In this approach, the people are asked how they look at and make meaning of their own mental health experience rather than importing meaning from somewhere else and imposing it on them. The IBH Model incorporates Filipino culture’s influence by nuancing content and method of mental health science based on indigenous perspectives.

### C. Social Determinants of Health

Health and wellbeing of people was once focused solely on medical care advancements. However, the glaring social factors that impact health cannot be overlooked. As the leading global authority on public health, the World Health Organization mirrored the historical development of the Social Determinants of Health (SDH) [15]. By the 1970s to 1980s, the organization published multiple studies to support the notion that social factors play a significant role in health. These studies reveal health inequities across different countries based on social factors and what can be done about them. As a result, the Commission on Social Determinants of Health (CSDH) was created. The CSDH final report on 2008 gave three recommendations: 1). interventions for children and their caregivers, creating healthy and safe spaces in the environment, improving employment conditions, social protection, and universal health care; 2) focus on improving economic and social conditions through progressive and systematic changes to power and wealth structures; 3) stakeholders from global leaders should pursue equity of health delivery to the grassroots. [16]. The IBH model incorporates the role of social determinants of health in diagnostics, therapeutics, and delivery of health care outcomes. It discusses the impact of poverty, unemployment, the lack of housing, the slum communities, the structures of wealth distribution, or lack thereof in pursuing mental health care delivery equity. [17].

### D. Emancipatory Pedagogy of Education

Emancipatory pedagogy is a theory in education introduced by Paulo Freire, a Brazilian educator who worked among non-literate populations. He states that there is an education that socializes people into what is taken to be an inherently oppressive society. Emancipatory education brings learners, teachers and educational practices to awareness of the different oppressive structures present in systems [18]. The role of the teacher is crucial to preserve the humanization and restore the dignity of every person that has been at stake as a result of these systems. The teacher enters a creative tension with students and the learning environment to confront oppressive structures in order to bring out a process of radical compassion that benefits not just a few, but everyone, especially the most vulnerable in society [19]. The IBH Model incorporates the principles of emancipatory education in its representation of the marginalized and vulnerable sectors in its content and process.

### E. Symptom Science

Symptom experiences of a psychological condition are molded by the individual’s histories and influenced by their relationships, often viewed in the sociology of health [20]. The perceptions of symptoms are generated from one’s innate experience, framed in the context of the individual’s interaction with the system present in the social environment. These perceptions of symptoms are complex because they can be understood from the point of view of the client, the clinician, and from context. The client’s perception of symptoms could be based on criteria such as meaningfulness of the experience, level of awareness of the experience, and the language used as a medium to express that experience. A medicalized model of symptoms that classify reported symptoms into categories like the DSM 5 [21] would miss out on the nuances of the patient-centered approach to understanding contexts of culture, developmental histories, neurological mechanisms, and other dimensions shed light to the psychological disorder. Symptom science takes a medical agnostic stance. It frees the reporting of experiences from the bias of a medical or psychiatric diagnosis. The IBH model takes an integrative approach applying a bio-psycho-social dimensional approach to symptom understanding. Clinicians and researchers who apply this approach would operationalize a client-centered understanding of disorders and outcomes from diagnostics to psychotherapeutics [22]. The IBH model necessitates a metatheoretical competency to critically analyze assumptions about the reality, truth, and objectivity of symptom experiences [23].

## II. METHOD

The authors performed a literature review based on the faculty consultations to identify the relevant themes related to an applied mental health concerns in clinical psychology, guided by the question, “What is a health psychology as a discipline that is holistic in its approach to mental health care delivery?” Initial consultations were sought with the academic institution’s psychology faculty. A series of the consultation was conducted with the department head and the assistant dean in social science, the teaching faculty members. Questions used for discussion were centered on identifying psychological needs that health psychologists could address in various locations like hospitals, schools, clinics, and primary care centers in *barangays* (local and rural communities). Based on the themes extracted from the literature review and peer discussions, the primary author (RB) designed a health psychology course that specifically addressed the mental health needs related to the COVID-19 pandemic. An action research was conducted as part of the class requirement to pilot the constructs identified in the literature search. The study specifically asked to answer the question: “What is the impact of social determinants of health and psychological distress on college students enrolled in an online learning course during the COVID-19 pandemic?” An assessment on the conduct and content of Health Psychology course was conducted.

## III. RESULTS

The anchor words from the literature search, the syllabus in health psychology given during COVID-19 pandemic, and the pilot research result output on the impact of social determinants and psychological disorders, based on the literature review, comprised the Integrative Behavioral Health (IBH) Model: 1. Emancipatory Education; 2) Filipino Psychology; 3) Contextualization; 4.) Philippine Mental Health Law; 5). Symptom Science; and 6) Social Determinants of Health (SDH). The pilot health psychology course content included a pilot research on the impact of social determinants of health and psychological distress on college students in online learning during COVID-19. Tables I and II summarize the findings of the study.

The following social determinants of health factors were examined in Table I. While the list of SDH factors are numerous, the research posited that these factors may have a possible relationship with the psychological distress of online learning students.

Table II presents a summary of the correlation test results within the same study. It would appear from the data that living with family members was negatively associated with being ready for online learning.

### A. The Conduct of Health Psychology Classes

The course was offered from April 25 to June 27, 2020 as an elective in a graduate school of psychology program at a private institution in southern Philippines. The course description stated the educational, scientific, and professional contribution of psychology to health, specifically designed to address the current COVID19 pandemic in the Philippine communities. Specifically, at the end of the course, the learners were expected to: a). Understand the etiology, promotion, and maintenance of health during and after the COVID 19 pandemic; b) Prevent, diagnose, treat, and rehabilitate physical and psychological disorders, c). Analyze psychological, social, emotional, and behavioral factors in physical and psychological disorders; d). Survey the current Philippine mental health care system and formulate mental health policies; e). Identify the gaps in clinical practice and research of the practice of clinical health psychology in the Philippines and design solutions to resolve the gaps. The class sessions lasted for ten meetings, with three hours every session in a webinar format. The following were the topics covered in the class: 1) Foundations of Clinical

Health Psychology; 2) Psychological Distress During COVID-19 Pandemic; 3) Theoretical Frameworks in Diagnoses, Assessment, and Interventions; 4) The IBH Model; 5) The Philippine Mental Health Law; 6) Primary Behavioral Health Care Model; 7) Action Research workshops; 8) Lifestyle Medicine and Integrative Medicine in Mental Health Model; 9) Addictive Behaviors During a Pandemic; 10) Psycho-Social Model in Oncology; 11) Psychological Care For Communities of Davao, Philippines Resources During COVID 19 Pandemic- A Virtual Symposium; 12) Class *fiesta* and celebration. The class three-hour webinar format began with a personal check-in of sharing highlights and low moments followed by a review and presentation of insights from the readings based on the homework given a week prior to the class meeting. The instructor (RB) introduced a new topic using the forms of didactics and case analyses. Homework was mostly done by submitting a summary of the readings and insights gathered from them. Partial reading is found in Appendix A.

## IV. DISCUSSION

### A. The Integrative Behavioral Health (IBH) Model

**Figure 1.**
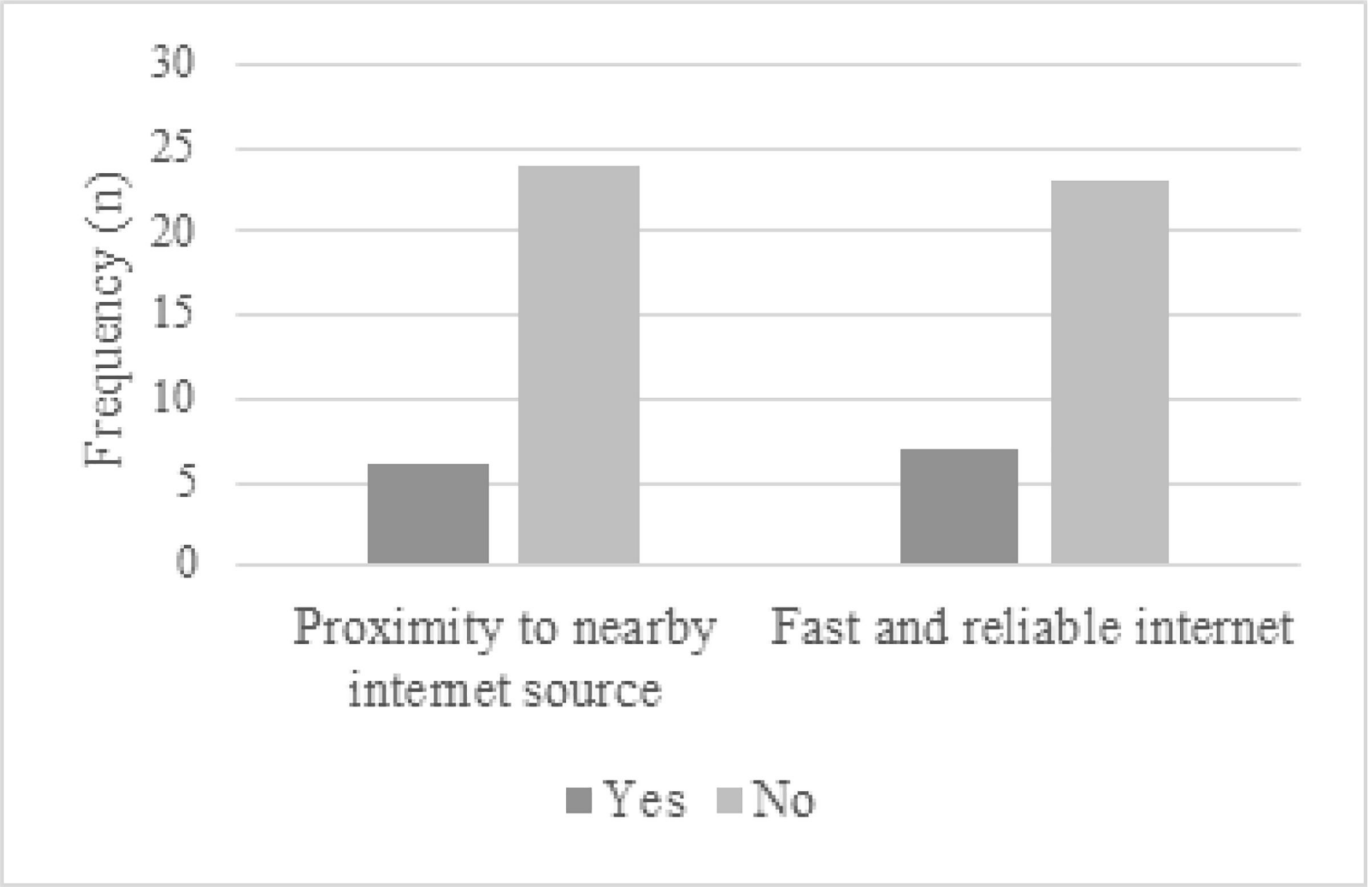
Access and quality of internet services of students taking online classes

The model proposes six components applied in four phases for curriculum development in health psychology discipline. Phase One applies the emancipatory pedagogy in the conduct of learning sessions. The teacher addresses social justice issues such as structural oppression that impact people’s mental health and observed during a crisis like COVID-19 pandemic. An emancipatory educator would, for example bring specific cases to the session where structural oppression exists like the corruption allegations of Philippine health insurance corporation amid COVID-19 and its impact to the social suffering of the citizens [24], the impact of stigma of sexual minorities like trans women who are being assaulted inside drug rehab [25] or killed [26], poverty as social suffering impacting the mental health resulting from the current pandemic [27], and other biosocial factors impacting equity of delivery of mental health care [28]. Structural misuse of power and its impact to health care deliveries, proposals to create systems for just implementations of mental health care in the grassroots are addressed through emancipatory pedagogy of education [29] using the Philippine Mental Health Law as the legal framework [30]. In Phase Two, the IBH Model identifies applies contextualization using the framework of *Sikolohiyang* Filipino and the guidelines of symptoms science in bio-psycho-social approach to client’s symptoms understanding and outcomes. Phase Three is the creation of subject areas that are essential to address the gaps of students, faculty, and the health psychology as a discipline. This phase of curriculum design focuses on faculty development and student formation towards their professional practice as clinicians. Delivery of mental health care is evidenced by their formation and faculty development. These are the ethical and professional delivery of care in mental health, the materiality of caring, and their presence in contexts where suffering is felt most. Both faculty and students demonstrate the value of care, versus the volume of care that can be lost in the commercialization of the helping profession [31], [32]. And finally, the morality of care where providers are in solidarity with the client. They will not leave the client until healing has taken place. The cultural value of *bayanihan* (burden bearing) is demonstrated in this phase. During the pandemic, the health psychology course taught by author (RB) also became a platform to embody the meaning of “We are in this together. I will be here with you, however long this will take all of us to recover”, slogan in an online teaching platform. Relationship which as cultural value of Filipinos also humanized the learning experience by starting every class with a check in. Every person present was to locate oneself during the pandemic, provide a number to identify the level of one’s distress, allow each person to unload stories of concern. The instructor was also conscious of identifying from the members who might be referred for further professional assistance. Solidarity of collective suffering during the pandemic has also made it possible for every person in class, including the instructor to draw strength from each other, to get inspiration from each other’s resilience, and to choose to hope during the time of the pandemic. The course culminated in a fiesta. It was to tap to the value of celebration of goodness of the experience in sessions. It was also a foresight of being able to have a reunion in the future after COVID-19 is over. The participants of the health psychology class affirmed that recovery from the pandemic will include the hope of communal fiestas and celebrations. Finally, in Phase Four, an audit is conducted anchored on the IBH model anchor words. Research on the translation of curriculum design to systems implementation of equity in the mental health care services to different context will be designed as an expected output. Further validation of metrics created as bases of assessment and evaluation will be produced. See Fig. 1 for context of services.

### B. Phase 1: Emancipatory Education, Philippine Mental Health Law

The Integrative Behavioral Health (IBH) Model takes into consideration the current social contexts where inequality exists due to social oppressive structures. The model also identifies the gaps in the implementation of the legislative policies and incorporates in the discipline social justice advocacies through policy making that will impact different aspects of its service delivery of care. An emancipatory education will also be intentional to reflect in the curriculum a system creation to lessen the chiasm and gap in the implementation of mental health care services in the grassroots. During COVID-19 pandemic, a health psychology course is intentional on how it can be more present in the materiality of care formed by the professional practice of psychology, but also the morality of care whereas professionals it is able to say in solidarity with the rest of the Filipino citizens, “We are in this suffering together. We will heal together.”

Looking for into the specifics of the mental health law that has implications to health psychology education and training, IBH proposes the following:

1. The need for an integrative approach/multi-disciplinary team approach

Provision of mental health services by mental health professionals to include medical doctors, psychologists, nurses, social workers or any other appropriately-trained and qualified professionals with relevant specific skills [33].

#### Implication:

This section of the law requires psychologists to work in a multi-disciplinary team in different healthcare settings like hospitals, schools, community primary health centers, and workplaces.

#### Gap:

The current health psychology education and curricula do not offer any orientation to train psychology students to function in a multi-disciplinary team in different health care delivery settings. The health psychology course or psychology education curricula, in general, have not saturated the different mental health care delivery settings such as hospitals, primary health care clinics in communities, workplaces, and schools.

- The framework of the Philippine mental health law has a strong leaning towards a medical model since the Department of Health, medical doctors head the government’s lead agency. In fact, the Philippine Mental Health Law defines a mental health condition along medicalized terms:

Mental health condition refers to a neurologic or psychiatric condition characterized by existence of a recognizable, clinically- significant disturbance in an individual’s cognition, emotional regulation, or behavior that reflects a genetic or acquired dysfunction in the neurological, psychosocial, or developmental processes underlying mental functioning. The determination of neurologic and psychiatric conditions shall be based on scientifically accepted medical nomenclature and best available scientific and medical evidence.

#### Implication:

The current training of health psychology students follows this leaning using a reductionist model of criteria-based diagnosis and the dependence of pharmacologic management of symptoms. The National Institutes of Mental Health introduced the Research Domain Criteria (R-Doc) to encourage the use of an evidence-based approach to mental health diagnoses and management [34]. For example, depression can be managed pharmacologically and by understanding its behavioral mechanisms based on neural circuitry, affect valence, attachment dynamics, motivation, cognitive functions, and the cultural practices in articulating these kinds of mood experiences expressions.

#### Gaps:

The R-DoC model as basis of evidence-based approach to psychological disorders is not being included in many existing psychology curricula. The use of medical terms for mental health conditions is helpful for case management within an American health care system like reimbursable insurance management plans that are driven by established psychiatric diagnoses. Hence, psychology needs to strengthen its research methodologies to build evidence showing its contribution in improving health outcomes using an alternative multi-disciplinary paradigm in providing mental health services.

- Mental health services in the community. Section 15, of Chapter 4 of the Mental Health Law mandates primary health care services to be integrated into the basic health services at the city, municipality, and barangay levels [33].

#### Implication:

In the health psychology training programs in the US, they use two models called Primary Care Behavioral Health (PCBH) and the Management for Patients with Mental Health Conditions (CoCM0) [35].

#### Gap:

Training models like PCBH have not been introduced or adapted in existing psychology curricula.

- Policy in the dissemination of Health Budgets. Chapter VI of the Mental Health Law requests capacity building and advancement of research and development.

This provision of the law allocates budgets to produce information, data, and evidence necessary to formulate and develop culturally relevant national mental health program incorporating indigenous concepts and practices related to mental health.

#### Implication:

The law acknowledges the role that academic institutions will play in the development of indigenous and culturally appropriate interventions.

#### Gap:

Psychology education has been wrestling since the late 1970s on how to indigenize the practice of psychology in the Philippines. The gap in the current health psychology program is to bring these methodologies in various healthcare settings.

The Integrative Behavioral Health Model Applied in Health psychology curriculum development.

#### Purposes:

**Figure 2.**
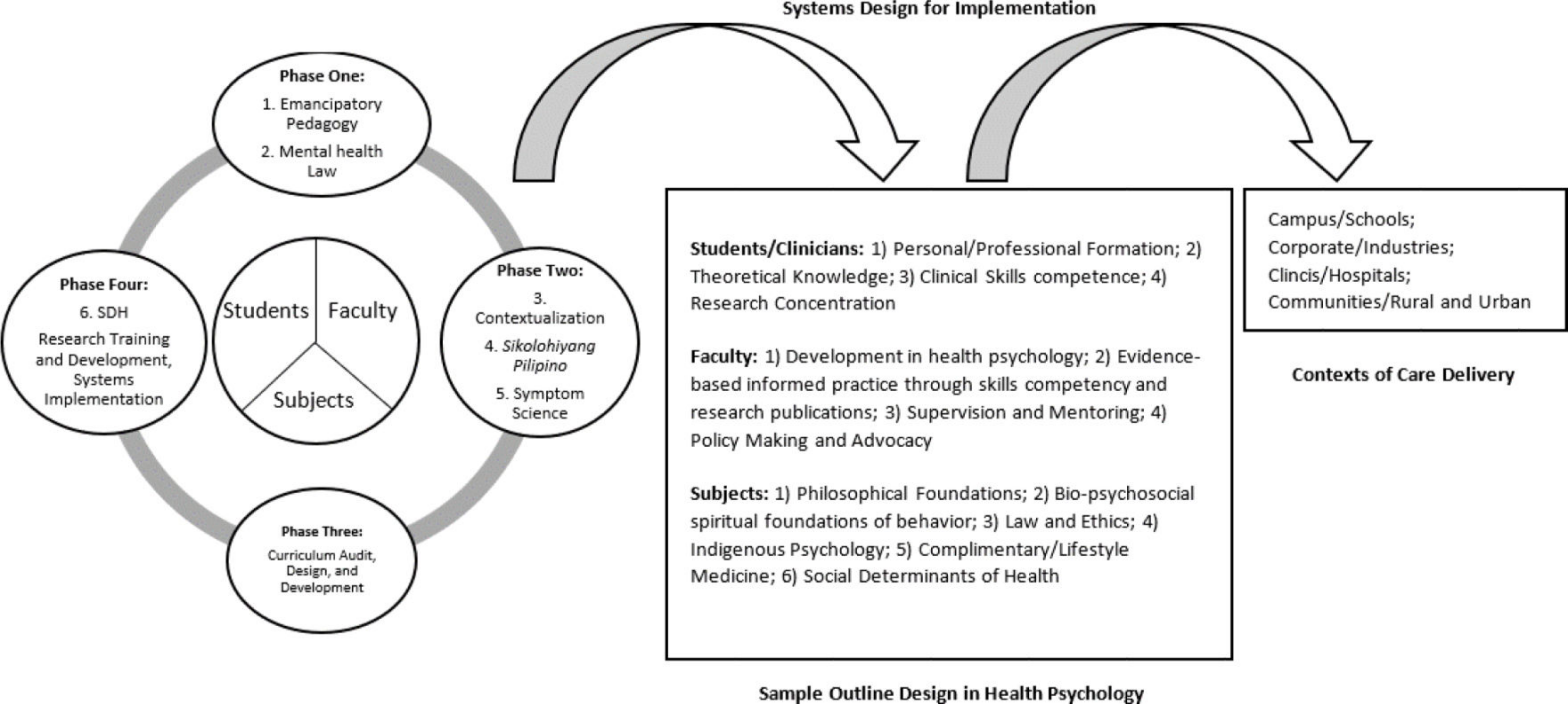
The implementation of Integrative Behavioral Health (IBH) model in health psychology curriculum

The IBH model will allow future psychologists to be significant key players in the implementation of the Philippine Mental Health Law. It will provide the framework for training future psychologists to address the gaps identified: the need to function in a multi-disciplinary team in various healthcare settings using culturally appropriate, evidence-based mental health services (See Fig. 2 for IBH Model).

### C. Phase 2: Contextualization, Filipino Psychology, Symptom Science

#### 1) Contextualization

In general, psychology is in the direction of moving towards evidence-based and evidence-informed practice in the delivery of mental health care in the Philippines. Core courses in sciences are incorporated in psychology curricula inspired by the need to develop evidence- based and evidence-informed practices. However, as a profession, it may not just be enough to enhance the curricula’ scientific content without understanding the soul of the context. Scientific content and context of culture in psychological studies should inform each other in a symbiotic dialogue, to navigate its creative tensions that can move the science of psychology forward, as well as promote cultural transformation through psychology. A ‘community to classroom’ approach of re-educating both providers and consumers of formalized information enhances knowledge acquisition [36]. The community members become informants to frame how knowledge is to be designed and applied. This is contextualization. Virgilio Enriquez envisioned a psychological practice where the understanding of symptoms, assessment of psychological conditions, its management and treatment should be at the core, sensitive to the experience of the Filipino [37] [38]. His strong commitment to contextualization was revealed when he asserted that the practice of psychology should be called, clinical psychology, but health psychology. He wanted to be sure that utmost respect to the Filipino *kaloob* (core of experience), is not lost in translation, in the name of scientific investigation informed from the western constructs [39]. This is where the constructs of Sikolohiyang Filipino may serve as the framework in the application and process of contextualization.

#### 2) Indigenous constructs’ intersection with behavioral health

The fundamental characteristic of the Filipino culture revolves around the constructs of *loob* (relational will) and *kapwa* (shared identity). As a relationship-oriented culture, Filipinos place emphasis on having harmonious relationships with the kapwa through the expression of *kagandahang loob* (beauty-of-will). The construct of loob is literally translated as “inside” [40]. This attempt to convert this construct to be accessible for foreign understanding misses the relational aspect of loob. When Filipinos talk about another person’s loob, they are often referring to that person’s intentions towards someone else [41]. Variants of the word loob, such as kagandahang loob (beauty-of-will) and *masamang loob* (evil person), pervade the language of Filipino characterization of others evincing its relational essence. Given the relational nature of the construct of loob, it then becomes inseparable to its object, the kapwa [42]. Often translated as “others”, this external perspective misses the point of the contribution of loob as both the direction and quality of the relationship to “others” [40]. That is, Filipinos consider those individuals who are their kapwa as the receiver of kagandahang-loob (in this case, “altruistic will”). The construct of kapwa then means someone whom Filipinos consider as not different from them, whom they share a common identity, and who is entitled to their altruistic intentions. The application of this construct towards mental health during COVID-19 pandemic is to also identify spaces of healing in the indigenous culture where collective relational experiences are resolved communally is a rich resource worthy of investigation. These are called *Agpulong* among Mangyan tribe in Central Philippines [43] are sources of hope and healing.

The Sikolohiyang Pilipino (Filipino Psychology) helps inform how the contextualization phase of IBH model is operationalized through the following exposures during internships students and research projects that could be collaboratively done with faculty members other investigators: 1) A patient-centered approach to understanding mental health symptoms; 2) Inclusions of complementary, integrative medicine and indigenous psychology practices in ethnic communities as foci of future researches; 3) Expand the barangay health center model to incorporate a primary behavioral health care; 4) A multi-disciplinary approach to primary behavioral health care to include clinical health psychologists; 5) Intentional training of allied mental health workers in the community who are lay people, overseen by committee members composed of multi-sectoral members and supervised by professional health psychologists, de-institutionalization of mental health systems by allowing a cooperative funded, participatory, multi-sectoral representatives of civil societies; 6) ‘Doctors to the Barrios’ programs will be adapted into ‘Psychologists to the Barrios’ as part of intentional internship in indigenous communities; 7) Incorporate Sikolohiyang Pilipino in indigenous research methodologies and scholarly contributions (e.g., publications, presentations) to center the focus on Filipino mental health experiences, allowing concepts and constructs being investigated in thesis and dissertations to be expressed in the mother tongue; 8) Inclusion of different indigenous worldviews that include the spiritualities of ethnic cultures as a modality for interventions; 9) Training of *datus* (community elders) and local leaders and community members as mental health care resources and providers.

### D. Phase 3: Curriculum, Design and Development

The IBH Model integrates a bio-psycho-social approach to symptoms science and understanding from diagnostics to psychotherapeutics. It also applies a multi-disciplinary approach to professional mental health care delivery. It is intentional in its creation of systems where the implementation of services is observed in the grassroots and different contexts where health disparities are observed. Table III is a proposed curriculum design that captures these elements of the IBH Model. The current drug war is one example of a gap between psychology as mental health discipline and the actual implementation of a mental health care delivery to address the problem. When Pres. Dueterte started the War on Drugs national campaign [44] psychologists as professionals were not at the leadership role to design a psychologically informed national mandate to implement a community-based drug intervention for drug abusers [45]. The psychology discipline has not included in its curricula addictions education to prepare future clinicians to address mental health problems with complex layers of economic, spiritual, political, and bio-behavioral etiologies, like addictions. IBH Model is intentional to begin curriculum design with an audit. The audit cascades from the institution’s mission/vision down to the health psychology program’s equity in delivering the mental health care services that are evidence informed, responsive to the current pandemic and beyond. Audit identifies the learning gaps of the learners as well as the competency gaps of teachers. The IBH model in health psychology curriculum is also cognizant of work in multi-disciplinary settings. Table III presents proposed elements of the curriculum design.

### E. Phase 4: Curriculum Audit, Research and Development

Implementation in the mental health delivery services is the bottle neck of health care. Learning institutions stay in the level of observation research, but not translating them to actual implementation of mental health care delivery. [31]. Equity in mental health care delivery is when most vulnerable are given the same equal chance of best care like the rest of its citizens [46].

Equity in mental health care delivery remains to be a challenge in the Philippines and worldwide. The main goal of Phase Four is on how systems of implementation for equity in the delivery of mental health services is achieved. A starting point could be on how SDH construct informs research agenda in systems for implementation. On the national level, the Philippines is partly trying to address in its 2016–2022 health agenda health inequity issues. Agenda set forth in the Philippine Health Agenda (PHA) include addressing social determinants of health such as urbanization, graying populations, climate change, and migration. What is further needed is generating evidence supporting this lens and utilizing these findings to mental health care deliveries via health psychology as a discipline. One of the fundamental elements of the system is the training, monitoring and supervision of local community members by the professional health psychologists to deliver the mental health care services in the grassroots and other vulnerable contexts. It has been given that there is a shortage of mental health professionals like health psychologists. In countries like Africa where there is one psychiatrist or psychologist per 1 million people, these countries created systems to address these concerns. The efficacy of interventions of trained lay counselors have been documented in countries like Fiji, Haiti, and Rwanda where the shortage of mental health professionals is significantly high [31].

## V. CONCLUSION

The COVID-19 pandemic has not only caused turmoil in terms of health and the economy but has also revealed flaws in the present social and mental health systems. Health inequity continues to be a detrimental factor in health outcomes and COVID-19 is not an exception. As professionals move past this pandemic, they must be more sensitive to context and adopt an interdisciplinary lens. Doing so fosters a calibrated decision-making in terms of addressing the gaps brought about by structural oppressive systems that must start in naming them in the learning sessions. Health psychologists translate the output of these curriculum design in equity in mental health care delivery as a form of social justice advocacy reflected in drafting social policies based on the legal framework of the National Mental Health Law of 2017. Post-COVID-19, clinical health psychologists and other stakeholders must elevate the voice of the unheard and empower underrepresented perspectives. The IBH curriculum model prepares future clinical health psychologists to be key members in a multi-disciplinary team to deliver an integrative approach to mental health service systems in developing economies like the Philippines. The model also provides the competencies required to provide comprehensive mental health services in the different settings such as the hospitals, the schools, and communities’ primary behavioral health centers, and the different work settings. Finally, the model achieves the vision of equity in mental health care delivery because professional health psychologists have incarnated themselves in the grassroots by creating systems of implementations that train, monitor and supervise local community members for the much needed mental health care necessary to recover from COVID-19 pandemic and beyond.

The COVID-19 pandemic has demonstrated the significance of a psychological and behavioral-science informed policymaking. Until a vaccine or a viable treatment has been completely deployed and administered, pandemic response relies on behavioral interventions instituted by national and local government units. The success of these behavioral interventions is rooted on understanding of how culture affects these health behaviors. Thus, the intersection of health psychology, curriculum development, and Sikolohiyang Filipino is more necessary now more than ever.

## Figures and Tables

**TABLE I.** The Social Determinants of Health Factors Used in the Study

Variable		Frequency	Percentage
Sex	Male	8	27.6%
	Female	21	72.4%
Area raised in urban setting	No	8	26.7%
	Yes	22	73.3%
Area of residence in urban setting	No	6	20.0%
	Yes	24	80.0%
Living with immediate or extended family member	No	5	16.7%
	Yes	25	83.3%
Sick for the last 6 months	No	16	57.1%
	Yes	12	42.9%
	Unsure	0	0.0%
With chronic health condition	No	26	86.7%
	Prefer not to say	4	13.3%
COVID case in City	No	1	3.4%
	Yes	28	96.6%
COVID case in sitio, purok, barangay	No	10	43.5%
	Yes	13	56.5%
Health Insurance	No	14	51.9%
	Yes	13	48.1%
Average family income	Prefer not to say	6	20.0%
	PHP 40,000 below	6	20.0%
	40,000 to 59,999	3	10.0%
	60,000 to 99,999	2	6.7%
	100,000 to 249,999	2	6.7%
	250,000 above	1	3.3%
	Unsure	10	33.3%
Means of Internet Access	Home internet services (DSL, Cable, Broadband)	18	
	Home internet services (DSL, Cable, Broadband), Mobile data services	11	
	Mobile phone data services	1	
Devices for Internet Access	Desktop PC, Laptop, Smartphone	1	
	Laptop	2	
	Laptop, Smartphone	22	
	Laptop, Tablet, Smartphone	1	
	Smartphone	4	

**TABLE II.** The Correlational Relationship of Social Determinants of Health on Psychological Distress of Students in Online Learning

Variable	Readiness for Online Learning	Psychological Distress
Sex	−.13	.17
Area raised in urban setting	.12	−.16
Area of residence in urban setting	.05	−.22
Living with family members	−.37*	.13
Being sick for the last six months	.2	.31
With chronic health condition/s	.04	.07
Covid-19 case present in your city	.01	−.14
Covid-19 case in your *sitio, purok, or barangay*	.1	−.33
Access to health insurance	−.01	0.14
Proximity to nearby internet source	.17	0.1
Fast and reliable internet	.32	0.2

* p < .05

**Table III.** Sample Curriculum Elements of Health Psychology

Subject Courses	Expected Student Outcomes	Teacher

**A. Bio-Behavioral Science**	**Knowledge Base**	- Refresher professional development in philosophical science (e.g. epistemology of naturalism and empiricism used in medical psychology, health psychology) - Strengthen faculty development in applied psychological science in areas of neuropsychology, psychopharmacology, and psychooncology, for example
Neuropsychology Psychopharmacology Health Psychology Neuropsychological Assessment Clinical Research Methods Behavioral Medicine Integrative Medicine	- Mastery of the biological basis of behavior - Analyze neural mechanisms of behaviors for specific disorders - Identify the chemical elements in the brain that impact behavioral change - Competency in experimental methods of research applied in clinical settings **Skills Development**	
**B. Social Science/Electives and Concentrations**	- Skills set in psychological first aid training of community mental health care workers	
*Sikolohiyang Pilipino* (Filipino Psychology) Social Determinants of Health Cognitive Therapy in Oncology Behavioral Primary Health Care Delivery Community-Based Interventions Addictive Behaviors Indigenous/Alternative Therapies	- Skills set in psychological first aid training of community mental health care workers - Research skills in quantitative methods - Competence in mixed methods research designs (qualitative and quantitative methods) - Competence in trauma-informed counseling and psychotherapy - Integrate research competence in Ecological Momentary Assessment Techniques in Clinical and Field Research - Appreciate the applications of a multidisciplinary team approach to symptoms management	- Training in new approaches of applied psychological science in primary behavioral health care in hospital and community-based settings - Faculty development in the areas of developing employees’ wellness programs as a model for maintaining quality of mental health in workplaces - Faculty being trained in designing mental health clinics in schools appropriate to the developmental stages of the school population - Faculty being trained in supervisory competence in hospital, school, community, and different work settings - Faculty development to include faculty as research specialists through local, national, and international research collaborations whose work and publications will be part of the bases of the Mental Health local and national service systems implementations. Their researchers will also serve ass bases for future policy making related to the effective implementation of the National Mental Health Law - Faculty development to include training in research grant writing applications
**C. Internships**	- Skills set in psychological first aid training of community mental health care workers	
Palliative and Hospice Care Units Hospitals Pediatric Oncology Units in Hospitals Traumatic Brain Injury Center in Mental Health Centers Trauma Play Centers for Children Barangay Behavioral Health Center for Family Wellness (Psychoeducation on Trauma from Disasters, Suicide Prevention, Depression, and Anxiety)	- Competence in the applications and validations of Filipino Medical Psychology methodologies of assessment, symptoms understanding and culturally sensitive management of mental health conditions - Training in evidence-based practices from assessment to management of medical and psychological conditions - Competence in medical and psychological ethics - Psychological First Aid	
	**Personal Professional Formation**	
D. Research (Thesis, Dissertation, and Collaborative Research Projects) Examples: Developmental pathways of Adolescent Substance Use Disorders - Whole Person Model to Psychosocial Cancer Palliative Care - The Effects of Mindfulness in Traumatic Brain Injury Symptoms	- Personal formation through receiving personal psychotherapy - Self-Care - Developing Personal Growth and Well Plan and Program - Clinical research competency	

## APPENDIX A PARTIAL READING LIST IN HEALTH PSYCHOLOGY

### A. Mental Health and the COVID-19 Pandemic

1. Public Mental Health Crisis during COVID-19 Pandemic, China
2. Bollinger, M. J., Hudson, T. J., Hu, B., Han, X., Long, C. R., & McElfish, P., 2020. The relationship between sociodemographic, behavioral, and clinical variables and pain in the Native Hawaiian and Pacific Islander population. Asian American Journal of Psychology, 11(1), 49–58. https://doi.org/10.1037/aap0000173
3. Bao, Y., Sun, Y., Meng, S., 2020. 2019-nCoV epidemic: address mental health care to empower society. https://doi.org/10.1016/S0140-6736(20)30309-3
4. Lai, J., Ma, S., Wang, Y., 2020. Factors Associated With Mental Health Outcomes Among Health Care Workers Exposed to Coronavirus Disease 2019. JAMA network open, 3(3), e203976. https://doi.org/10.1001/jamanetworkopen.2020.3976
5. Public Mental Health Crisis during COVID-19 Pandemic, China, 2020.
6. Wang, C., Pan, R., Wan, X., 2020. Immediate Psychological Responses and Associated Factors during the Initial Stage of the 2019. Coronavirus Disease (COVID-19) Epidemic among the General Population in China. International Journal of Environmental Research and Public Health
7. Tang, C., 2005. Psychosocial Factors Influencing the Practice of Preventive Behaviors against the Severe Acute Respiratory Syndrome Among Older Chinese in Hong Kong. Sage Publications
8. Dong L, Bouey J., 2020. Public mental health crisis during COVID-19 pandemic, China. Emergency. Infectious Diseases. https://doi.org/10.3201/eid2607.200407
9. Li, W., Yang, Y., Liu, Z., 2020. Progression of Mental Health Services during the COVID-19 Outbreak in China. International Journal of Biological Sciences.
10. Qui, J., Shen, B., Zhao, M., 2020. A nationwide survey of psychological distress among Chinese people in the COVID-19 epidemic: implications and policy recommendations

### B. World Mental Health

1. World Mental Health Action Plan 2013–2020 Manual
2. Keiler, R., 2004. Prevalence, Severity, and Unmet Need for Treatment of Mental Disorders in the World Health Organization World Mental Health Surveys. Journal of American Medical Association. (Retrieved April 2020)
3. Lund, C., Breen, A., Flisher, AJ., 2010. Poverty and common mental disorders in low and middle income countries: A systematic review. Social Science Medicine
4. Murray, C. 2018. Global, regional, and national incidence, prevalence, and years lived with disability for 354 diseases and injuries for 195 countries and territories, 1990–2017: a systematic analysis for the Global Burden of Disease Study 2017. Lancet Journal.
5. Anderon, P., Llopis, E., Hafsman,C.,2011. Reducing the burden of impaired mental health. Health Promotion International Journal.
6. The State of Mental Health in America, 2017 Manual

### C. Action Research

1. Sheppard, B., Hartwick, J., Warshaw, P., 1988. The Theory of Reasoned Action: A Meta-Analysis of Past Research with Recommendations for Modifications. Journal of Consumer Research.

### D. Integrative Medicine in Mental Health, Lifestyle Medicine

1. Gordon, N., Salmon, R., Wright, B., 2015. Clinical Effectiveness of Lifestyle Health Coaching: Case Study of an Evidence-Based Program. American Journal of Lifestyle Medicine.
2. Capaldi, E., 1996. Why we eat what we eat. The psychology of eating.

### E. Primary Behavioral Health

1. Peek. C. 2013. Lexicon for Behavioral Health and Primary Care Integration Concepts and Definitions Developed by Expert Consensus

### F. Foundations of Clinical Health Psychology

1. Melo, E., 2018 Emancipatory Education and Youth Engagement in Brazil: A Case Study Bridging the Theory and Practice of Education for Social Transformation. Education Science Journal
2. Freire, P. (2007). Pedagogy of the Oppressed. NY: Continuum.
3. Mental Health Act of 2017, Republic Act 110361, Codified and amended into National Mental Health Law on June 20, 2018. Congress of the Philippines. Republic of the Philippines. Manila.
